# Genome-Wide Identification of Hsp40 Genes in Channel Catfish and Their Regulated Expression after Bacterial Infection

**DOI:** 10.1371/journal.pone.0115752

**Published:** 2014-12-26

**Authors:** Lin Song, Jiaren Zhang, Chao Li, Jun Yao, Chen Jiang, Yun Li, Shikai Liu, Zhanjiang Liu

**Affiliations:** The Fish Molecular Genetics and Biotechnology Laboratory, Aquatic Genomics Unit, School of Fisheries, Aquaculture and Aquatic Sciences, Auburn University, 203 Swingle Hall, Auburn, AL 36849, United States of America; Instituto Butantan, Brazil

## Abstract

Heat shock proteins (HSPs) consist of a large group of chaperones whose expression is induced by high temperature, hypoxia, infection and a number of other stresses. Among all the HSPs, Hsp40 is the largest HSP family, which bind to Hsp70 ATPase domain in assisting protein folding. In this study, we identified 57 hsp40s in channel catfish (*Ictalurus punctatus*) through *in silico* analysis using RNA-Seq and genome databases. These genes can be classified into three different types, Type I, II and III, based on their structural similarities. Phylogenetic and syntenic analyses provided strong evidence in supporting the orthologies of these HSPs. Meta-analyses of RNA-Seq datasets were conducted to analyze expression profile of Hsp40s following bacterial infection. Twenty seven hsp40s were found to be significantly up- or down-regulated in the liver after infection with *E. ictaluri*; 19 hsp40s were found to be significantly regulated in the intestine after infection with *E. ictaluri*; and 19 hsp40s were found to be significantly regulated in the gill following infection with *F. columnare*. Altogether, a total of 42 Hsp40 genes were regulated under disease situations involving three tissues and two bacterial infections. The significant regulated expression of Hsp40 genes after bacterial infection suggested their involvement in disease defenses in catfish.

## Introduction

Molecular chaperones include a large and diverse group of proteins that assist in folding or unfolding of other proteins or macromolecule structures in cells [1]. Among these, heat shock proteins (HSPs) constitute the largest groups of chaperones. They were found to be induced under various stresses such as elevated temperature, hypoxia [2] and diseases [3], [4]. HSPs are classified based on their molecular weight to include Hsp105/110, Hsp90, Hsp70, Hsp60, Hsp40, Hsp10, and small HSPs (sHSPs) [5].

Hsp40 proteins, also known as DnaJ proteins, constitute one of the largest families among heat shock proteins. They regulate the ATPase activity of Hsp70 proteins whose function is reversibly binding to partially denatured protein substrates to avoid the aggregation of themselves or with other molecules [6]–[8]. In the Hsp70-Hsp40 co-chaperone system, the association between Hsp70 proteins and substrates requires an ATP binding to ATPase domain and then being hydrolyzed to change conformation of the binding domain. Thus, various Hsp70's substrates could specifically bind to its least conserved C-terminal at a higher affinity. However, because the ATPase activity of Hsp70s is extremely weak, The J-domain of Hsp40s is needed for activating the ATPase domain of Hsp70s [8], [9].

Hsp40s share a 70-amino acids conserved J-domain, similar to the 73-amino acid-domain of prototypical DnaJ protein in *Escherichia coli* [10]. The DnaJ of *E. coli* typically consists of four regions: N-terminus J-domain, glycine/phenylalanine-rich region, Cysteine repeats and variable C-terminus domain (CTD) [10]. According to the homology of the DnaJ protein of *E.coli*, Hsp40 proteins were classified into three types: Type I DnaJ proteins (DnaJA) possess all four parts of DnaJ protein in *E. coli*; Type II DnaJ proteins (DnaJB) possess the N-terminus J-domain and the glycine/phenylalanine-rich region; Type III DnaJ proteins (DnaJC) only have a J-domain, which is not necessarily located at N-terminus of the protein [11], [12]. Recently, type IV DnaJ protein family was added, which differs from the other three types of DnaJ proteins in that it owns a ‘J-like’ domain [6], [13], [14] containing various mutations in a highly conserved histidine, proline, and aspartic acid–HPD motif located between helices II and III in DnaJ domain [6], [15]–[17]. However, Peter Walsh et al. (2004) proposed that the term J-proteins should be used more strictly to describe only J proteins with well-conserved J-domain in the HPD motif, while structurally less-conserved proteins should be referred to as J-like proteins [6].

Although heat shock proteins are traditionally regarded as being induced by heat and stresses, recent studies suggested HSPs may actually play important roles in immune responses [18]. For example, HSPs are considered to mediate humoral and cellular innate immune responses [19]; HSPs in extracellular environment serve as a danger signal to activate innate immune cells such as dendritic cells and macrophages [20]–[22]. Several cytokines can be induced by HSPs, including TNF-α, IL-1β, IL-12, nitric oxide and some chemokines [23]; HSPs can also stimulate adaptive immune responses as potent antigen carriers. Hsp60, Hsp70, Hsp90 have been reported to interact with immune cells as a ligand for a variety of cell-surface receptors such as Toll-like receptors [25], [26] and a number of CDs such as CD14 and CD91 [24]–[27].

Due to Hsp40 and Hsp70 worked together as a co-chaperone, the immune function especially the expression fold change trend of this two proteins should be taken into consideration at the same time. In teleost, hsp70 genes have been found to be involved in bacterial kidney disease in coho salmon [28] and vibriosis in rainbow trout [29]. In olive flounder, Hsp40 proteins were found to be up-regulated in flounder embryonic cells (FEC) after viral infection and a flounder hsp70 gene was also expressed in heat-shocked and virus treated FEC cells [30], indicating hsp40 and hsp70 functioned as co-chaperone in antiviral immune responses. In the kidney of olive flounder, *dnaja4, dnajb6* and *dnajb11* were found to be expressed after being infected by *Streptococcus parauberis* [31]. However only limited studies have been done on the roles of Hsp40s in disease resistance.

RNA-Seq-based expression analysis has become a robust method to assess transcriptional profile to different challenge experiments [32]. In our recent RNA-Seq studies, we have successfully obtained comprehensive transcriptome assemblies from catfish intestine and liver after *E. ictaluri* infection and from catfish gill after *F. Columnare* infection [33]–[35]. The expression patterns of differentially expressed genes from these three studies were validated by quantitative real-time RT-PCR with average correlation coefficient around 0.9 (p<0.001).

Channel catfish (*Ictalurus punctatus*) is the leading aquaculture species in the United States. Its genomic resources have been well developed in recent years, particularly ESTs [36], transcriptome sequences generated by RNA-Seq [37], [38] and draft whole genome sequence (unpublished data). These resources make it feasible to conduct systematic analysis of hsp40 genes in channel catfish genome. The objective of this study was to determine the involvement of hsp40 genes in disease responses after bacterial infection in catfish. Here we report the genome-wide identification of a full set of 57 hsp40 genes, their phylogenetic and syntenic analyses, and their involvement in disease responses after bacterial infection with ESC and columnaris using RNA-Seq datasets.

## Materials and Methods

### Database mining and sequence analyses

In order to identify the full set of hsp40 genes in channel catfish, we collected all Hsp40 proteins from teleost fishes (zebrafish, three-spined stickleback, medaka, tilapia and fugu) and other species (human, mouse, platypus, chicken, turtle and frog) (S1 Table). These sequences were retrieved from NCBI (http://www.ncbi.nlm.nih.gov) and Ensembl (http://www.ensembl.org) databases and used as queries to search against channel catfish RNA-Seq datasets. The e-value was set at intermediately stringent level of e-10 for collecting as many as potential hsp40-related sequences for further analysis. The retrieved sequences were then translated using ORF finder (http://www.ncbi.nlm.nih.gov/gorf/gorf.html). Further, the predicted ORFs were verified by BLASTP against NCBI non-redundant (Nr) protein sequence database. The simple modular architecture research tool (SMART) [39] was used to predict the conserved domains based on sequence homology and further confirmed by conserved domain prediction from BLAST. The predicted catfish Hsp40s proteins and all other query sequences were utilized to search against catfish genome database using TBLASTN program. The retrieved genome scaffolds were then predicted by FGENESH in SoftBerry (http://linux1.softberry.com/berry.phtml?topic=fgenesh&group=programs&subgroup=gfind).

### Phylogenetic and conserved syntenic analyses

All the amino acids from channel catfish and other species were used to construct phylogenetic tree. Multiple protein sequences alignments were conducted using the Clustal W2 program [40] and MUSCLE 3.8 [41]. Three alignment methods: L-INS-i, E-INS-i and G-INS-i were applied from MAFFT 7.01 [42] and the best alignment with highest score was evaluated by program MUMSA [43]. JTT+I+G model [Jones-Taylor-Thornton (JTT) matrix incorporated a proportion of invariant sites (+I) and the gamma distribution for modeling rate heterogeneity (+G)] was selected as the best-fit model by ProtTest 3 program [44] according to the Bayesian information criterion. Maximum likelihood phylogenetic tree was constructed using MEGA5.2.2 [45] with bootstrap test of 1,000 replicates. Final phylogenetic tree was separated into three different phylogenetic trees according to classification of subfamilies due to the large size.

Conserved syntenic regions surrounding the relevant hsp40 genes were searched by examining the conserved co-localization of neighboring genes on scaffold of channel catfish and other species based on genome information from Ensembl (Release 74) and NCBI database. Neighbor genes of channel catfish Hsp40 genes were predicted by FGENESH [46] and BLASTP.

### Meta-analysis of expression of hsp40 genes and bacterial challenge

The Illumina-based RNA-Seq reads were retrieved from bacterial challenge experiments in catfish: intestine samples challenged with *Edwardsiella ictaluri* (SRA accession number SRP009069) [34], liver samples challenged with *E. ictaluri* challenge (SRA number SRP028159) [33] and gill samples challenged with *Flavobacterium Columnare* (SRA number SRP012586) [35]. Trimmed high-quality reads were mapped onto the catfish Hsp40 genes using CLC Genomics Workbench software (version 5.5.2; CLC bio, Aarhus, Denmark). Mapping parameters were set as ≥95% of the reads in perfect allignment and ≤2 mismatches. The total mapped reads number for each transcript was determined and normalized to analyze RPKM (Reads Per Kilobase of exon model per Million mapped reads). The proportions-based Kal's test was performed to identify the differently expressed genes comparing with control sample and fold changes were calculated. Transcrirps with absolute fold change value ≥1.5, *p*-value ≤0.05 and total read number ≥5 were included in the analyses as significantly differently expressed genes.

## Results

### Identification of Hsp40 genes in catfish

A total of 57 Hsp40 genes were identified in channel catfish. Their classification, domain structures, and GenBank accession numbers are summarized in Table 1. Type I included 6 Hsp40 genes; type II included 16 Hsp40 genes; and type III included 35 subfamily C Hsp40 genes. Among all these genes, almost all sequences were identified in both transcriptome and genome databases with full-length except *dnajb9* with partial sequences in both databases. These catfish hsp40 genes were named following Zebrafish Nomenclature Guidelines (https://wiki.zfin.org/display/general/ZFIN+Zebrafish+Nomenclature+Guidelines).

**Table 1:** Summary of 57 *Hsp40* genes identified in the catfish genome.

Name	Type	ORF	Domain Structure	Accession number
***dnaja1***	I	complete	DnaJ-CXXCXGXG-DnaJ_C	JT413966
***dnaja2***	I	complete	DnaJ-CXXCXGXG-DnaJ_C	JT408916
***dnaja2l***	I	complete	DnaJ-CXXCXGXG-DnaJ_C	JT412411
***dnaja3a***	I	complete	DnaJ-CXXCXGXG-DnaJ_C	JT425696
***dnaja3b***	I	complete	DnaJ-CXXCXGXG-DnaJ_C	JT410497
***dnaja4***	I	complete	DnaJ-CXXCXGXG-DnaJ_C	JT340875
***dnajb1a***	II	complete	DnaJ-DnaJ_C	JT410595
***dnajb1b***	II	complete	DnaJ-DnaJ_C	JT405623
***dnajb2***	II	complete	DnaJ-3*(UIM)	JT413231
***dnajb4***	II	complete	DnaJ-DnaJ_C	JT418024
***dnajb5***	II	complete	DnaJ-DnaJ_C	JT410284
***dnajb5L***	II	complete	DnaJ-DnaJ_C	JT407526
***dnajb6a***	II	complete	DnaJ	JT280415
***dnajb6b***	II	complete	DnaJ	JT477624
***dnajb9L1***	II	complete	DnaJ	JT406977
***dnajb9L2***	II	complete	DnaJ	JT244156
***dnajb9***	II	Partial	DnaJ	JT383860
***dnajb11***	II	complete	DnaJ-DnaJ_C	JT410155
***dnajb12a***	II	complete	DnaJ-DUF1977	JT411397
***dnajb12b***	II	complete	DnaJ-DUF1977	JT348699
***dnajb13***	II	complete	DnaJ-DnaJ_C	JT413284
***dnajb14***	II	complete	DnaJ-DUF1977	JT417082
***dnajc1***	III	complete	DnaJ-SANT	JT407463
***dnajc2***	III	complete	(DnaJ)-2*(SANT)	JT342108
***dnajc3(prkri)***	III	complete	7*(TPR)-DnaJ	JT407497
***dnajc3***	III	complete	7*(TPR)-DnaJ	JT410821
***dnajc4***	III	complete	DnaJ	JT400171
***dnajc5ab***	III	complete	DnaJ	JT412549
***dnajc5aa***	III	complete	DnaJ	JT483994
***dnajc5gb***	III	complete	DnaJ	JT406264
***dnajc6***	III	complete	(PTPc_DSPc)-DnaJ	JT410375
***dnajc7***	III	complete	7*(TPR)-DnaJ	JT278330
***dnajc8***	III	complete	DnaJ	JT278586
***dnajc9***	III	complete	DnaJ	JT223126
***dnajc10***	III	complete	DnaJ-4*(Thioredoxin)	JT424718
***dnajc11***	III	complete	DnaJ-DUF3395	JT406478
***dnajc12***	III	complete	DnaJ	JT411276
***dnajc13***	III	complete	DnaJ	JT411283
***dnajc14***	III	complete	DnaJ	JT340805
***dnajc15***	III	complete	DnaJ	JT417797
***dnajc16***	III	complete	DnaJ-Thioredoxin	JT405377
***dnajc16L***	III	complete	DnaJ-Thioredoxin	JT413761
***dnajc17***	III	complete	DnaJ-RRM_1	JT411855
***dnajc18***	III	complete	DnaJ-DUF1977	JT414201
***dnajc19***	III	complete	DnaJ	JT348880
***dnajc20(hscb)***	III	complete	DnaJ-HSCB_C	JT410908
***dnajc21***	III	complete	DnaJ-ZnF_U1- ZnF_C2H2	JT414736
***dnajc22***	III	complete	TM2-4*(Transmembrane)- DnaJ	JT405460
***dnajc23(sec63)***	III	complete	DnaJ-Sec63	JT409443
***dnajc24***	III	complete	DnaJ-CSL zinc finger	JT407343
***dnajc25***	III	complete	DnaJ	JT406198
***dnajc26(gak)***	III	complete	S_TKc-STYKc-PTPc_DSPc-PTEN_C2-STYKc-DnaJ	JT418500
***dnajc27***	III	complete	Small GTPase-DnaJ	JT464820
***dnajc28***	III	complete	DnaJ-DUF1992	JT413325
***dnajc29(sacs)***	III	complete	UBQ-2*(HATPase_c)-DnaJ-HEPN	JT399814/JT345237
***dnajc30a***	III	complete	DnaJ	JT399756
***dnajc30b***	III	complete	DnaJ	JT199100

**Figure 1:**
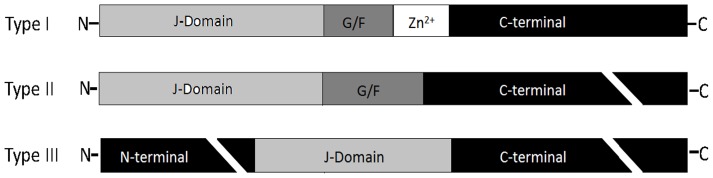
Schematic presentation of three sub-types of HSP40 family. Type I DnaJ proteins have full domain conservation with *Escherichia coli* DnaJ, type II DnaJ proteins have a J domain and G/F (Gly/Phe-rich region) motif at N-terminus, type III DnaJ proteins only have a J domain anywhere in the protein.

Six type I genes were identified in the catfish genome including *dnaJa1, dnaJa2, dnaJa2-like, dnaJa3a, dnaJa3b* and *dnaJa4*. These Hsp40 genes are homologous to DnaJ of *E.coli*, whose structure is conservatively comprised of N-terminal J-domain, glycine/phenylalanine-rich region, cysteine repeats motif and variable C-terminus domain (CTD) (Fig. 1 and Table 1).

Sixteen type II genes were identified in channel catfish including *dnaJb1a, dnaJb1b, dnaJb2, dnaJb4, dnaJb5, dnaJb5-like, dnaJb6a, dnaJb6b, dnaJb9, dnaJb9-like1, dnaJb9-like2, dnaJb11, dnaJb12a, dnaJb12b, dnaJb13 *.and *dnaJb14*., of which dnaJb1 was among the most highly heat-inducible HSPs in human [12].

A total of 35 type III hsp40 genes were identified from the catfish transcriptome and confirmed with the genome database including *dnaJc1, dnajc2, dnajc3, dnajc3 (prkri), dnajc4, dnajc5aa, dnajc5ab, dnajc5gb, dnajc6, dnajc7, dnajc8, dnajc9, dnajc10, dnajc11, dnajc12, dnajc13, dnajc14, dnajc15, dnajc16, dnajc16-like, dnajc17, dnajc18, dnajc19, dnajc20 (hscb), dnajc21, dnajc22, dnajc23 (sec63), dnajc24, dnajc25, dnajc26 (gak), dnajc27, dnajc28, dnajc29 (sacs), dnajc30a* and *dnajc30b*. Catfish type III Hsp40 proteins only have one J-domain in their structure, which is not necessarily located at N-terminus of the protein (Fig. 1 and Table 1). Orthologies were established for all the type III Hsp40s among human, zebrafish and catfish. However, several type III Hsp40s, i.e., *Dnajc3 (Prkri), Dnajc20 (Hscb), Dnajc23 (Sec63), Dnajc26 (Gak),* and *Dnajc29 (Sacs)* have not been annotated as DnaJC members. They are currently named according to aliases of human DNAJC proteins respectively [12].

### Phylogenetic analysis of channel catfish Hsp40s

A total of 57 channel catfish Hsp40 genes have been phylogenetically analyzed. Each type of Hsp40 was subsequently analyzed separately (S1–S5 Figs.). Type III is divided into three parts due to its enormous size of the phylogenetic tree (S3–S5 Figs.). In a few cases where it was difficult to establish orthologies due to duplications (*dnajb9, dnajb12* and *dnajc30*), syntenic analyses were also conducted (see below). We renamed some of the ambiguous names of hsp40 genes from other fish according to their relationship with the relevant zebrafish genes on the phylogenetic tree. The phylogenetic trees were then reconstructed after standardizing all the names. In the phylogenetic tree, all the members of catfish Hsp40 were well distributed into distinct clades and grouped with corresponding genes of zebrafish and other fishes, which were supported by strong bootstrap value (S1–S5 Figs.).

### Syntenic analysis of channel catfish Hsp40s

**Figure 2:**
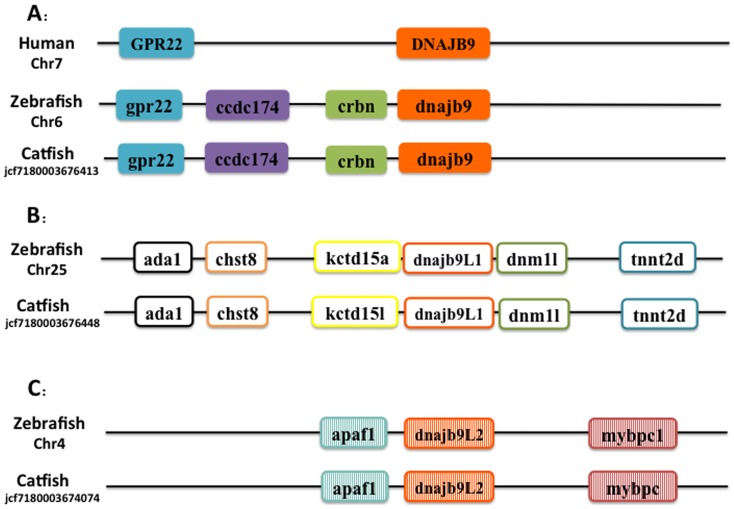
Schematic presentation of the conserved synteny blocks neighboring Dnajb9 gene (A) Dnajb9-like1 gene (B) and Dnajb9-like2 gene (C). Note that in each case, the gene order and orientation was relatively conserved. Abbreviations: (A) GPR22, G Protein Receptor 22; Dnajb9, Dnaj (Hsp40) homolog, subfamily B, member 9; ccdc174, Coiled-Coil Domain Containing 174; crbn, cereblon. (B) ada1, Adenosine Deaminase; chst8, carbohydrate (N-acetylgalactosamine 4–0) sulfotransferase 8; kctd15a, potassium channel tetramerisation domain containing 15a; dnajb9L1 DnaJ (Hsp40) homolog, subfamily B, member 9 like 1; dnm1l, dynamin 1-like; tnnt2d, troponin T2d, cardiac. (C) apaf1, apoptotic protease activating factor 1; dnajb9l2 DnaJ (Hsp40) homolog, subfamily B, member 9 like 2; mybpc, myosin binding protein C.

Though phylogenetic relationships provide strong support for the identities of most Hsp40 genes, syntenic analyses were required to provide additional evidence for orthologies or otherwise the paralogies for several hsp40 genes including the duplicated hsp40 genes such as *dnajb9, dnajb12 and dnajc30*. Positions of these catfish hsp40 genes and their neighbor genes were identified from the draft genome scaffolds. And the genes were also identified from the zebrafish genome. As shown in Fig. 2, three *dnajb9*-related genes were analyzed. The gene with the highest level of conservation in gene contents and gene orders as compared with the human DNAJB9 was named *dnajb9* in zebrafish (accession number from ensembl: ENSDARP00000094644). Two other genes similar to *dnajb9* in zebrafish with NCBI accession number: NP_001020355.1 and NP_001019564.1 were named *dnajb9L1* and *dnajb9L2* (L refers to “like”) respectively. All those genes of catfish, as well as those related genes from other fish species, were therefore named as *dnajb9L1* and *dnajb9L2* accordingly.

**Figure 3:**
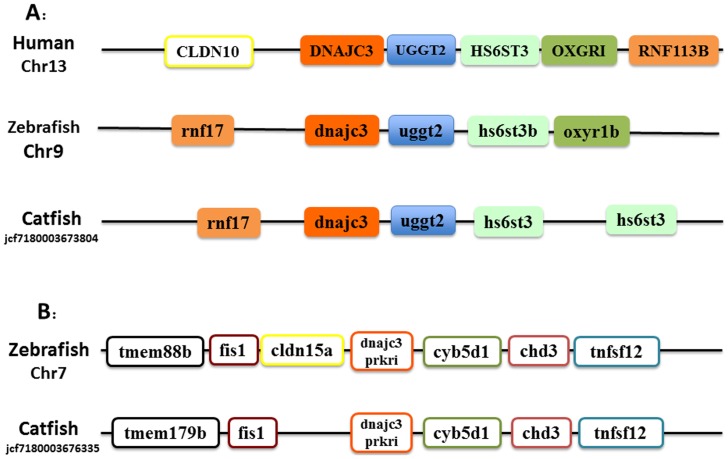
Schematic presentation of the conserved synteny blocks neighboring DnaJc3 (A) and DnaJc3-prkri gene (B). Note that in each case, the gene order and orientation was relatively conserved. Abbreviations: (A) CLDN10, claudin 10; rnf17, ring finger protein 17; DnaJc3, DnaJ (Hsp40) homolog, subfamily C, member 3; uggt2, UDP-glucose glycoprotein glucosyltransferase 2; hs6st3b, heparan sulfate 6-O-sulfotransferase 3b; oxyr1b, oxoglutarate (alpha-ketoglutarate) receptor 1b; RNF113B, ring finger protein 113B. (B) tmem88b, transmembrane protein 88 b; fis1, fission 1 (mitochondrial outer membrane) homolog (S. cerevisiae); cldn15a, claudin 15a; dnajc3 prkri, protein-kinase, interferon-inducible double stranded RNA dependent inhibitor; cyb5d1, cytochrome b5 domain containing 1; chd3, chromodomain helicase DNA binding protein 3; tnfsf12, tumor necrosis factor (ligand) superfamily, member 12.

As shown in Fig. 3, of the two *dnajc3*-related genes, the neighboring genes surrounding the DNAJC3 gene is clearly well conserved among human, zebrafish and catfish (Fig. 3A), suggesting their orthologies. However, the genes surrounding the other *dnajc3*-related gene were quite different between the fish genes and the human genes (compare Fig. 3A and Fig. 3B), suggesting that they were not orthologous. However, the genes surrounding this gene are well conserved between zebrafish and catfish. We therefore annotated this genes as *prkri* as annotated in zebrafish.

**Figure 4:**
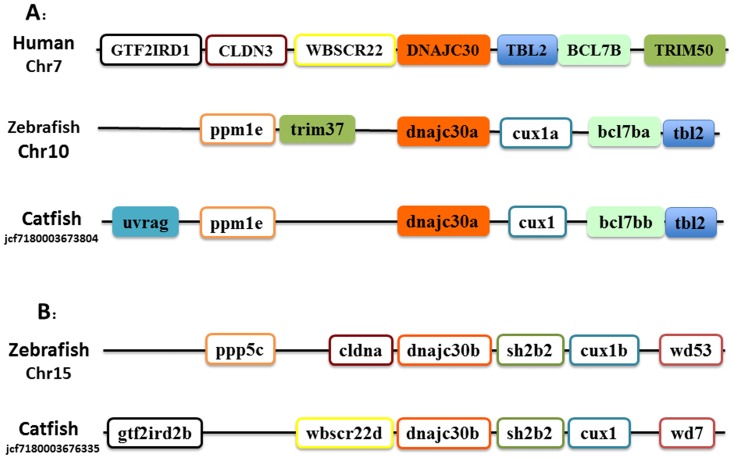
Schematic presentation of the conserved synteny blocks neighboring DnaJc30a (A) and DnaJc30b gene (B). Note that in each case, the gene order and orientation was relatively conserved.Abbreviations: (A) GTF2IRD1, GTF2I repeat domain containing 1; CLDN3, claudin 3; WBSCR22, Williams Beuren syndrome chromosome region 22; DnaJc30, DnaJ (Hsp40) homolog, subfamily C, member 30; TBL2, transducin (beta)-like 2; BCL7B, B-cell CLL/lymphoma 7B; TRIM50, tripartite motif containing 50; ppm1e, protein phosphatase 1E (PP2C domain containing); trim37, tripartite motif containing 37; cux1a, cut-like homeobox 1a; uvrag, UV radiation resistance associated. (B) ppp5c, protein phosphatase 5, catalytic subunit; cldna, claudin a; dnajc30b, DnaJ (Hsp40) homolog, subfamily C, member 30; sh2b2, fission 1 (mitochondrial outer membrane) homolog (S. cerevisiae); wd53, WD repeat domain 53; wd7, WD repeat domain 7; gtf2ird2b, GTF2I repeat domain containing 2b.

Two *dnajc30*-related genes were found in catfish and zebrafish. As shown in Fig. 4, these two genes were located on two different chromosomes of zebrafish, but genes surrounding the duplicated gene are well conserved among human, zebrafish and catfish, suggesting that they were derived from the whole genome duplication event. Therefore, they were annotated as *dnajc30a* and *dnajc30b*, following zebrafish gene nomenclature system.

### Hsp40s copy number variation among species

Catfish have almost all the orthologues of Hsp40s in human and zebrafish (Table 2), with exception of several genes existing in humans, but absent from teleost fishes including *Dnajb3*, *Dnajb7,* and *Dnajb8.* It is interesting that quite a few of the Dnaja genes and Dnajb genes have duplicated copies in various teleost species, however, most of the Dnajc genes have only a single copy in the teleost genomes (Table 2). Specifically, *Dnaja2*, *Dnaja3, Dnajb1, Dnajb5, Dnajb6, Dnajb12, Dnajc3, Dnajc7, Dnajc11, Dnajc16* and *Dnajc30* were found to have two duplicates, and *Dnajb9* was found to have three copies in catfish and most of the teleost fish while only one copy was found in other species. Note that *Dnajc5* have been found to have five copies in zebrafish, three copies in catfish and three copies in human as well. This is the only gene that has more than one copy in the human genome. Compared to zebrafish, channel catfish has fewer copies for *Dnajb12, Dnajc5, Dnajc7 and Dnajc11* (Table 2).

**Table 2:** Comparison of copy numbers of HSP40 genes among selected vertebrate genomes.

	Gene	Human	Chicken	Frog	Zebrafish	Catfish	Medaka	Tilapia	Fugu
**Type I**	***Dnaja1***	1	1	1	1	1	1	1	0
	***Dnaja2***	1	1	1	2	2	2	2	2
	***Dnaja3***	1	1	1	2	2	2	2	2
	***Dnaja4***	1	1	2	1	1	1	1	1
	**Subtotal**	**4**	**4**	**5**	**6**	**6**	**6**	**6**	**5**
**Type II**	***Dnajb1***	1	0	1	2	2	2	2	2
	***Dnajb2***	1	1	1	1	1	1	1	1
	***Dnajb3***	1	0	0	0	0	0	0	0
	***Dnajb4***	1	1	1	1	1	1	1	1
	***Dnajb5***	1	1	1	2	2	2	2	3
	***Dnajb6***	1	1	2	2	2	2	2	2
	***Dnajb7***	1	0	0	0	0	0	0	0
	***Dnajb8***	1	1	0	0	0	0	0	0
	***Dnajb9***	1	1	1	3	3	2	3	1
	***Dnajb11***	1	1	1	1	1	2	1	1
	***Dnajb12***	1	1	1	3	2	2	2	2
	***Dnajb13***	1	1	1	1	1	1	1	1
	***Dnajb14***	1	1	1	1	1	2	1	1
	**Subtotal**	**13**	**10**	**11**	**17**	**16**	**17**	**16**	**15**
**Type III**	***Dnajc1***	1	1	1	1	1	1	1	1
	***Dnajc2***	1	1	1	1	1	1	1	1
	***Dnajc3***	1	1	1	2	2	2	2	2
	***Dnajc4***	1	0	1	1	1	1	1	1
	***Dnajc5***	3	1	2	5	3	2	1	2
	***Dnajc6***	1	1	1	1	1	1	1	1
	***Dnajc7***	1	1	1	2	1	2	2	2
	***Dnajc8***	1	1	1	1	1	1	1	1
	***Dnajc9***	1	1	1	1	1	1	1	1
	***Dnajc10***	1	1	1	1	1	1	1	1
	***Dnajc11***	1	1	1	2	1	2	2	2
	***Dnajc12***	1	1	1	1	1	1	1	1
	***Dnajc13***	1	1	1	1	1	1	1	1
	***Dnajc14***	1	1	1	1	1	1	1	1
	***Dnajc15***	1	1	1	1	1	1	1	0
	***Dnajc16***	1	1	1	2	2	2	2	2
	***Dnajc17***	1	1	1	1	1	1	1	1
	***Dnajc18***	1	1	1	1	1	1	1	1
	***Dnajc19***	1	1	1	1	1	1	1	1
	***Hscb(Dnajc20)***	1	1	1	1	1	1	1	1
	***Dnajc21***	1	1	1	1	1	1	1	1
	***Dnajc22***	1	1	1	1	1	1	1	1
	***Dnajc23***	1	1	1	1	1	1	1	1
	***Dnajc24***	1	1	1	1	1	1	1	1
	***Dnajc25***	1	1	1	1	1	1	1	1
	***Dnajc26***	1	1	1	1	1	1	1	1
	***Dnajc27***	1	1	1	1	1	1	1	1
	***Dnajc28***	1	1	1	1	1	1	1	1
	***Dnajc29***	1	1	1	1	1	1	1	1
	***Dnajc30***	1	1	1	2	2	1	1	2
	**Subtotal**	**32**	**29**	**31**	**39**	**35**	**35**	**34**	**35**
	**Total**	**49**	**43**	**47**	**62**	**57**	**58**	**56**	**55**

### Regulated expression of hsp40 genes in catfish after bacterial infection

**Figure 5:**
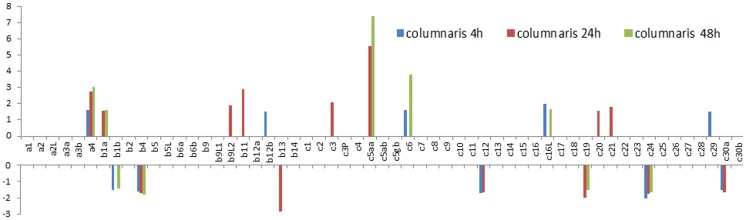
Column bar chart showing the fold change of Hsp40s expression in *F. Columnare* challenge experiments. Vertical axis shows the value of fold change. Gene names are shown as clipped name without “dnaj”. For instance, a1 means “*dnaja1*”. “c3P” represents “*dnajc3_prkri*”.

Using three bacterial challenged RNA-Seq datasets (intestine sample infected by *E. ictaluri*, liver sample infected by *E. ictaluri,* and intestine sample infected by *F. columnare*), the involvement of hsp40 genes after bacterial infection was determined. As shown in S2 Table, 42 out of a total of 57 hsp40s were involved in disease defense responses. Specifically, 19 hsp40s were found regulated in the gill following *F. columnare* infection. Among them, 12 genes were up-regulated (1.5× fold change cutoff) and 7 genes were down-regulated (1.5× fold change cutoff) after columnaris infection (Fig. 5). Among these regulated hsp40 genes, some of them are transiently up- or down-regulated while others are gradually induced or suppressed. For instance, Dnajb9L2, Dnajb11, Dnajb12b, Dnajc3, Dnajc20, Dnajc21, and Dnajc29 were up-regulated at only one time point (1.5× fold change cutoff); similarly, Dnajb13 was only down regulated at 24h after infection. In contrast, Dnaja4, Dnajb1a, Dnajc5aa, Dnajc6, and Dnajc16L were up-regulated in at least two time points after infection, suggesting their up-regulated expression was more lasting. Similar patterns were observed for down-regulated genes including Dnajb1b, Dnajb4, Dnajc12, Dnajc19, Dnajc24, and Dnajc30a (Fig. 5).

**Figure 6:**
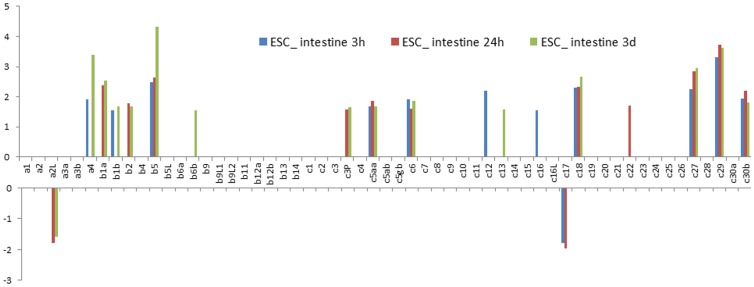
Column bar chart shows the fold change of Hsp40s expression in intestine after *E. ictaluri* challenge experiments. Vertical axis shows the value of fold change. Gene names are shown as clipped name without “dnaj”. For instance, a1 means “*dnaja1*”. “c3P” represents “*dnajc3_prkri*”.

A total of 19 hsp40 genes were found to be regulated in the intestine after *E. ictaluri* infection. Among these, 17 were up-regulated while two were down-regulated. The upregulated hsp40 genes included Dnaja4, Dnajb1a, Dnajb1b, Dnajb2, Dnajb5, Dnajb6b, prkri, Dnajc5aa, Dnajc6, Dnajc12, Dnajc13, Dnajc16, Dnajc18, Dnajc22, Dnajc27, Dnajc29, and Dnajc30b. The two down-regulated hsp40 genes were Dnaja2L, and Dnajc17. Significantly regulated expression of most of these genes were observed in more than one time points with exception of Dnajb6b, Dnajc12, Dnajc13, Dnajc16, and Dnajc22 where the significantly regulated expression was observed at only one time point (Fig. 6).

**Figure 7:**
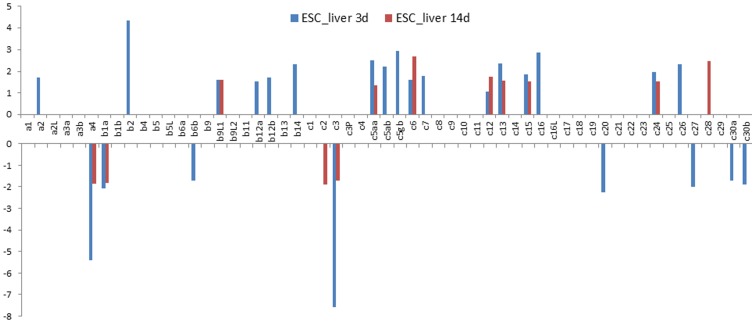
Column bar chart shows the fold change of Hsp40s expression in liver after *E. ictaluri* challenge experiments. Vertical axis shows the value of fold change. Gene names are shown as clipped name without “dnaj”. For instance, a1 means “*dnaja1*”. “c3P” represents “*dnajc3_prkri*”.

While RNA-Seq datasets for the gill tissue after columnaris infection and the intestine tissue after ESC infection were from early infection period of 4 h to 3days after infection, we analyzed RNA-Seq datasets from liver tissues collected at 3 days and 14 days after infection. As shown in Fig. 7, a total of 27 hsp40 genes were found to be regulated during this period in the liver. It is apparent that many of the hsp40 genes were induced at only one time points at 3 days or 14 days after infection, but not at both time points. For instance, Dnaja2, Dnajb2, Dnajb12a, Dnajb12b, Dnajb14, Dnajc5ab, Dnajc5gb, Dnajc7, Dnajc16, and Dnajc26 were up-regulated only at 3 days after infection, but not at 14 days after infection; similarly, Dnajc28 was up-regulated only at 14 days after infection, but not at 3 days after infection. Very similarly, of the 9 down-regulated genes, six were regulated at only one time point. Down-regulated expression was observed mostly at 3 days after infection as the up-regulated hsp40 genes, but not at 14 days after infection. Only Dnajc2 was found to be down-regulated 14 days after infection in the liver tissues (Fig. 7).

## Discussion

Hsp40 proteins play key roles in assisting protein folding by activating the ATPase domain of Hsp70. In spite of their importance, only a few Hsp40 genes were characterized from channel catfish [47]. Systematic analysis of channel catfish Hsp40 genes has been lacking. In this study, we identified a full set of 57 catfish Hsp40 genes in the catfish genome. This was achieved by thorough analysis of rich genomic and transcriptomic resources including several hundred thousands of ESTs [33]–[35], RNA-Seq transcriptome assemblies [37], [38], and the draft genome sequences (unpublished).

Phylogenetic and syntenic analyses allowed annotation of Hsp40 genes. It is apparent that catfish harbored most of hsp40 genes. Compared with the human genome, catfish lacked only three hsp40 genes, similar to the situation of other teleost fish species. However, a number of paralogues were also discovered in catfish due to duplication. The phylogenetic analysis strongly supported the nomenclature of catfish hsp40 genes in all three subfamilies. Most Hsp40s are conserved through evolution, while there are still special preferences made by teleost and catfish. First, the teleost tended to have more duplications than mammals, likely as a consequence of whole genome duplication. Among various fish species, *DnaJa2, DnaJa3, DnaJb1, DnaJb5, DnaJb9, DnaJb12, DnaJc3, DnaJc7, DnaJc11, DnaJc16,* and *DnaJc30* were found to be duplicated while in mammals, birds and amphibians only one copy of these genes were found.

Meta-analysis of disease challenges revealed a gene fold change profile of catfish hsp40s involving in disease defense and stress protection. As mentioned in the introduction, although regulated expression of HSPs after infection have been reported in several fish species, systematic analysis of their involvement in diseases has not been conducted. This work, therefore, represents the first systematic analysis of Hsp40 involvement after bacterial infection among all species.

**Figure 8:**
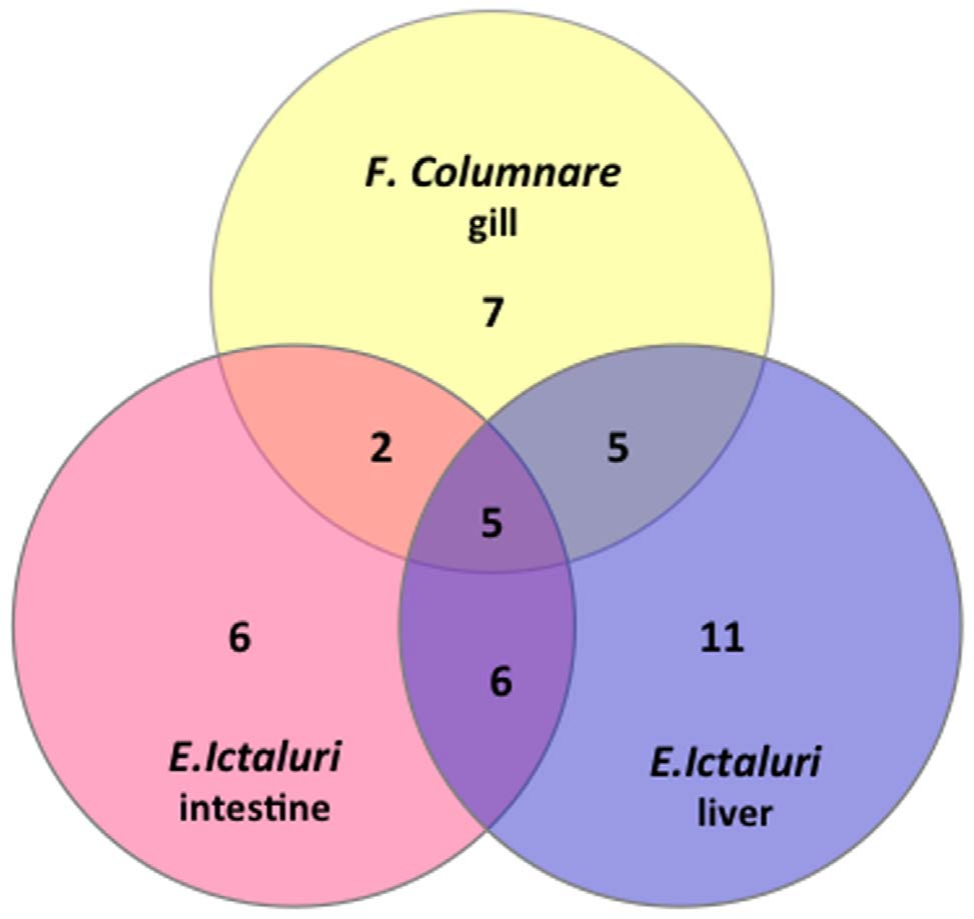
Venn diagram of catfish Hsp40 gene expression showing overlapped pattern of induced expression under various challenge conditions.

Surprisingly, a large number of hsp40 genes, 42 in total, were up- or down- regulated after bacterial infection, suggesting their extensive involvement in disease responses. Specifically, a total of 27 hsp40 genes were found up- or down- regulated in liver after *E. ictaluri* infection, 19 genes were found regulated in intestine after *E. ictaluri* infection, and 19 genes were found regulated in gill following *F. columnare* infection (Fig. 8). Of these 42 regulated hsp40 genes, 5 genes: *dnaja4, dnajb1a, dnajc5aa, dnajc6 and dnajc12* were regulated in all three situations, suggesting their importance as general disease response hsps; 7 genes (*dnajb1b*, *dnajc29, dnaja4, dnajb1a, dnajc5aa, dnajc6 and dnajc12*) were commonly regulated with columnaris infection in the gill and ESC infection in the intestine; 10 genes (*dnajb12b, dnajc3, dnajc20, dnajc24, dnajc30a, dnaja4, dnajb1a, dnajc5aa, dnajc6 and dnajc12)* were commonly regulated with columnaris infection and ESC infection in the liver; and 11 genes (*dnajb2, dnajb6b, dnajc13, dnajc16, dnajc27, dnajc30b, dnaja4, dnajb1a, dnajc5aa, dnajc6 and dnajc12)* were commonly regulated with infection of ESC in the intestine and ESC infection in the liver (Fig. 8).

In spite of a set of commonly regulated hsps, disease- and tissue-specific genes in each challenge experiment were observed. For instance, after *F. columnare* infection, seven genes (*dnajb4, dnajb9L2, dnajb11, dnajb13, dnajc16L, dnajc19* and *dnajc21)* were found to be specifically regulated in the gill. Similarly, six hsp40 genes (*dnaja2L, dnajb5, prkri, dnajc17, dnajc18* and *dnajc22*) were found to be specifically regulated in the intestine after ESC infection; and 11 hsp40 genes (*dnaja2, dnajb9L1, dnajb12a, dnajb14, dnajc2, dnajc5ab, dnajc5gb, dnajc7, dnajc15, dnajc26* and *dnajc28)* were found to be specifically regulated in the liver, suggesting their specific roles in a tissue- and time-dependent manner.

The vast majority of hsp40 genes were found to be significantly regulated soon after infection at 3h or 24 h after infection with both columnaris and ESC, suggesting their involvement in the early phases of disease response. Only *dnajb6b and dnajc13* were not significantly up-regulated until 3 days after infection. However, in the liver at late stages of disease development, more hsp40 genes (nine) were found to be down-regulated. These findings could be explained by the co-chaperon system of Hsp40 and Hsp70 [48], [49]. Hsp70 regulates the intracellular function and fate of proteins through the formation of direct protein-protein interactions that occur largely through an EEVD-binding domain in its C terminus [50]–[52]. In innate immune system, Hsp70 can serve as the endogenous ligand of TLR2 and TLR4 and aid to recognize the bacteria [53]. Previous work demonstrated that TLRs were up-regulated at 1 h post challenge [54] by *F. columnare* and most up-regulated at 6 h and 24 h post challenge by *E. ictaluri* [55] in catfish. It is believed that since Hsp70 serves as a TLR4 agonist, there is a positive correlation between HSP70 and TLR4 in human. Therefore, the increased or decreased fold of Hsp40s after infection of bacteria can reflect the fold change of its co-chaperone Hsp70 and thus the associated TLRs [56], [57]. Furthermore, it was also reported that TLRs are down-regulated after 36 h. This could be the explanation to the large number of down-regulated in liver 3 days and 14 days post challenge from our meta-analysis. It is interesting to observe that seven genes were down-regulated with columnaris whereas only two genes were down-regulated with ESC at early stages of disease development after infection. This observation indicated that the immune response to the *F. columare* was faster than that to *E. ictaluri*. In general, hsp40 genes were up-regulated at early stages of diseases, and with time, they tended to be down regulated as the diseases progressed. In the same line of thoughts, columnaris disease may involve more rapid destructive processes than ESC diseases and therefore, more hsp40 genes were found to be down-regulated even at early stages of columnaris disease development (Fig. 5).

The level of regulated expression varied among the genes as well as tissues and time after infection. For most of the hsp40 genes, up- or down-regulation was less than five-fold as compared with the controls. Only three genes were regulated more than five times after bacterial infections. Dnajc5aa was up-regulated 6-8 times after columnaris infection (Fig. 5). No hsp40 genes were up- or down-regulated more than 5-fold in the intestine after ESC infection. However, in the liver, and Dnaja4 and Dnajc3 were down-regulated more than five-fold in the liver after infection with ESC.

## Conclusions

We have identified and annotated a full set of 57 hsp40 genes. The vast majority (42 out of 57) of hsp40 genes were either up- or down-regulated after infection. Their patterns of regulation were tissue-, time- and disease-specific, but it appeared that at earliest stages of disease infections, the majority of hsp40 genes were up-regulated whereas with the progression of the diseases, more and more hsp40 genes became down-regulated.

## Supporting Information

S1 Fig **Phylogenetic tree of Hsp40s Type I.** The phylogenetic tree was constructed by Mega5.2.2 using the Maximum Likelihood method based on the JTT matrix-based model of amino acid substitution as described in detail in Material and Method section. Numbers around the nodes correspond to bootstrap support values in percentages. A discrete Gamma distribution was used to model evolutionary rate differences among sites (5 categories (+G, parameter  = 1.1981)). The rate variation model allowed for some sites to be evolutionarily invariable ([+I], 2.8567% sites). All positions with less than 95% site coverage were eliminated. That is, fewer than 5% alignment gaps, missing data, and ambiguous bases were allowed at any position. There were a total of 332 positions in the final dataset. Accession numbers for all protein sequences used in the analysis are provided in S1 Table. The black dots indicate catfish dnaja genes. Suffix “L” indicated “-like”, for instance, dnaja2L means dnaja2-like.
(TIF)

S2 Fig **Phylogenetic tree of Hsp40s type II.** The phylogenetic tree was constructed as in S1 Fig. Accession numbers for all sequences are provided in S1 Table. Suffix “L” indicated “-like”, for instance, dnajb5L means Dnajb5-like.
(TIF)

S3 Fig **Phylogenetic tree of Hsp40s type III: Dnajc1 to Dnajc9.** The phylogenetic tree was constructed as in S1 Fig. Accession numbers for all sequences are provided in S1 Table. The black dots indicate catfish Dnajc genes. Suffix “L” indicated “-like”, for instance, Dnajc7L means Dnajc7-like.
(TIF)

S4 Fig **Phylogenetic tree of Hsp40s type III: Dnajc10 to Dnajc20.** The phylogenetic tree was constructed as in S1 Fig. Accession numbers for all sequences are provided in S1 Table. The black dots indicate catfish Dnajc genes. Suffix “L” indicated “-like”.
(TIF)

S5 Fig **Phylogenetic tree of Hsp40s type III: Dnajc21 to Dnajc30.** The phylogenetic tree was constructed as in S1 Fig. Accession numbers for all sequences are provided in S1 Table. The black dots indicate catfish Dnajc genes. Suffix “L” indicated “-like”.
(TIF)

S1 Table **Gene names and accessions of reference Hsp40s used in this study.**
(XLSX)

S2 Table **Fold changes of Hsp40s under each challenge.** Bold font is the significant expression.
(XLSX)
