# Supplementary figures and images for: Genome-Wide Identification of Hsp40 Genes in Channel Catfish and Their Regulated Expression after Bacterial Infection
Source: PLoS One. 2014 Dec 26;9(12):e115752. doi: 10.1371/journal.pone.0115752 (PMC4277396; doi:10.1371/journal.pone.0115752)

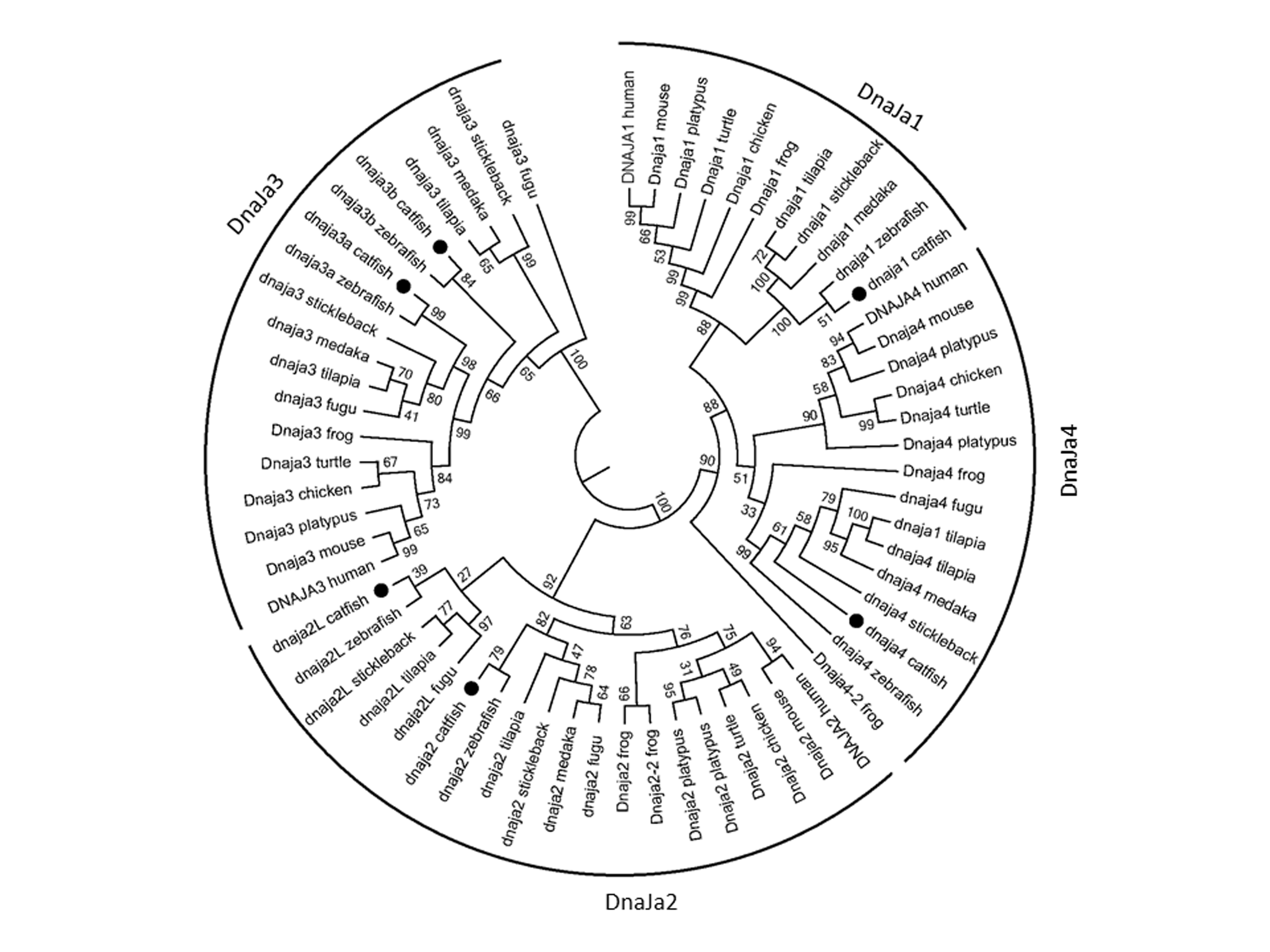

Supplement: S1 Fig — Phylogenetic tree of Hsp40s Type I. The phylogenetic tree was constructed by Mega5.2.2 using the Maximum Likelihood method based on the JTT matrix-based model of amino acid substitution as described in detail in Material and Method section. Numbers around the nodes correspond to bootstrap support values in percentages. A discrete Gamma distribution was used to model evolutionary rate differences among sites (5 categories (+G, parameter = 1.1981)). The rate variation model allowed for some sites to be evolutionarily invariable ([+I], 2.8567% sites). All positions with less than 95% site coverage were eliminated. That is, fewer than 5% alignment gaps, missing data, and ambiguous bases were allowed at any position. There were a total of 332 positions in the final dataset. Accession numbers for all protein sequences used in the analysis are provided in S1 Table. The black dots indicate catfish dnaja genes. Suffix “L” indicated “-like”, for instance, dnaja2L means dnaja2-like. (TIF) [file pone.0115752.s001.tif]

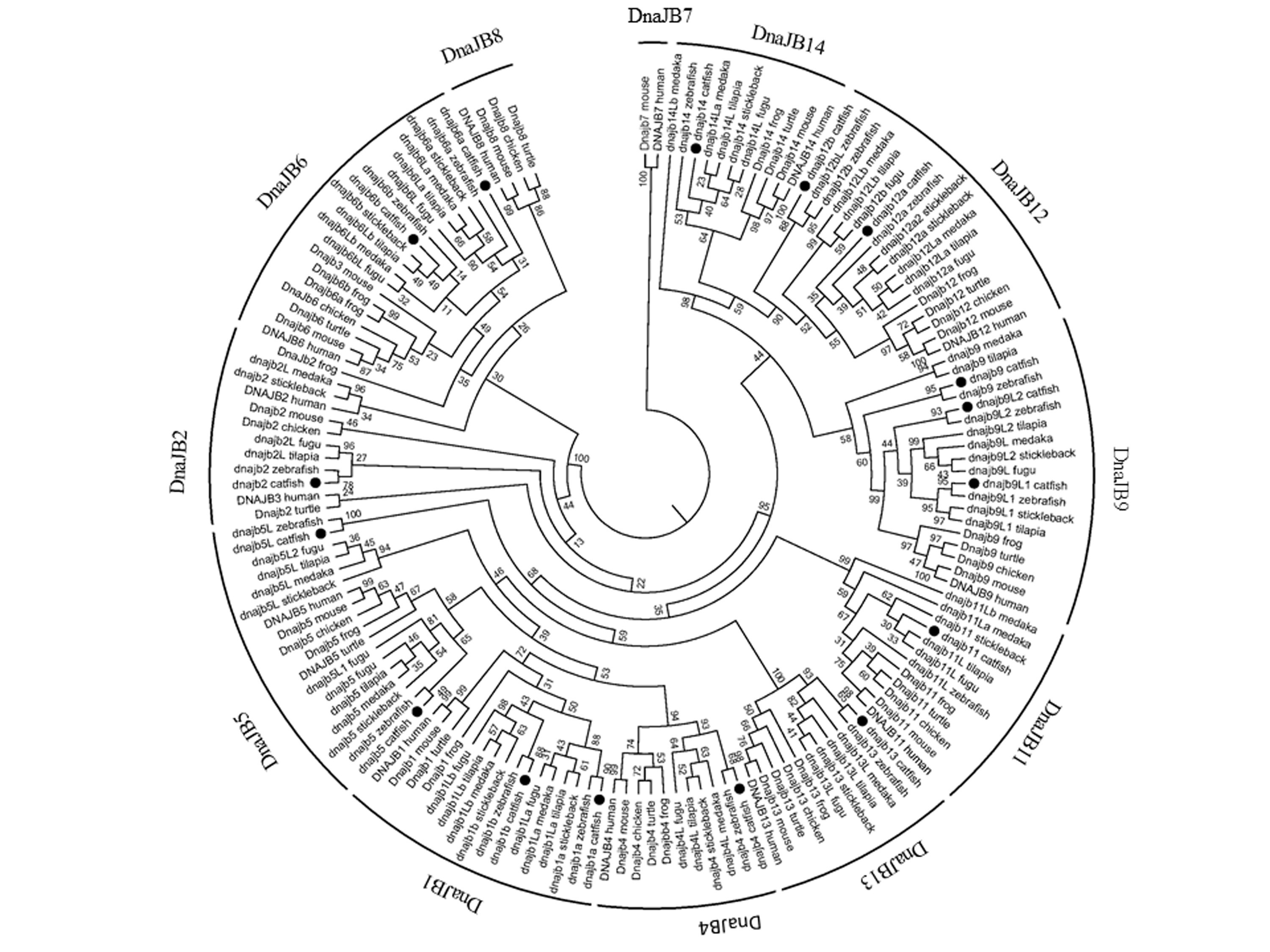

Supplement: S2 Fig — Phylogenetic tree of Hsp40s type II. The phylogenetic tree was constructed as in S1 Fig. Accession numbers for all sequences are provided in S1 Table. Suffix “L” indicated “-like”, for instance, dnajb5L means Dnajb5-like. (TIF) [file pone.0115752.s002.tif]

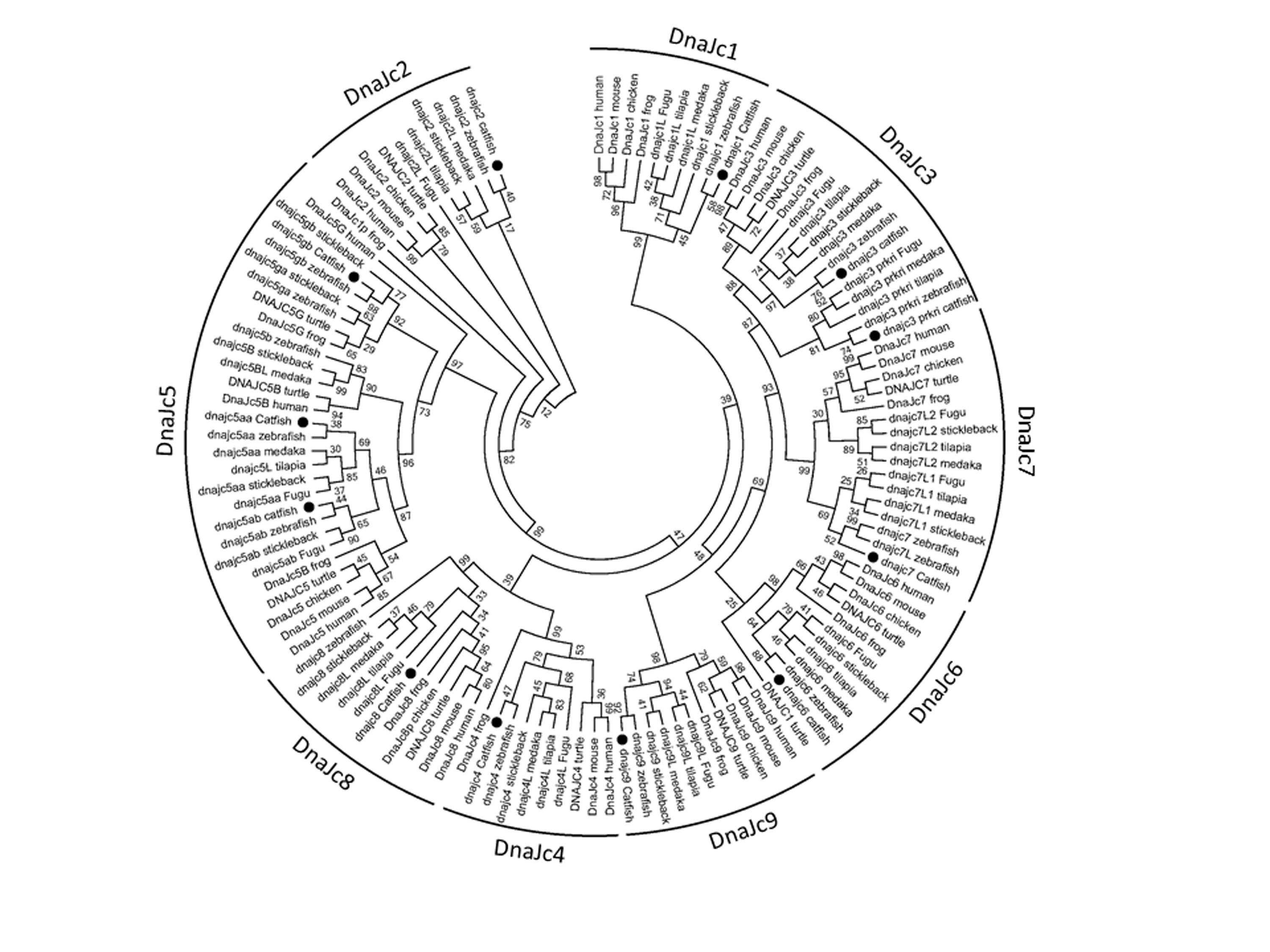

Supplement: S3 Fig — Phylogenetic tree of Hsp40s type III: Dnajc1 to Dnajc9. The phylogenetic tree was constructed as in S1 Fig. Accession numbers for all sequences are provided in S1 Table. The black dots indicate catfish Dnajc genes. Suffix “L” indicated “-like”, for instance, Dnajc7L means Dnajc7-like. (TIF) [file pone.0115752.s003.tif]

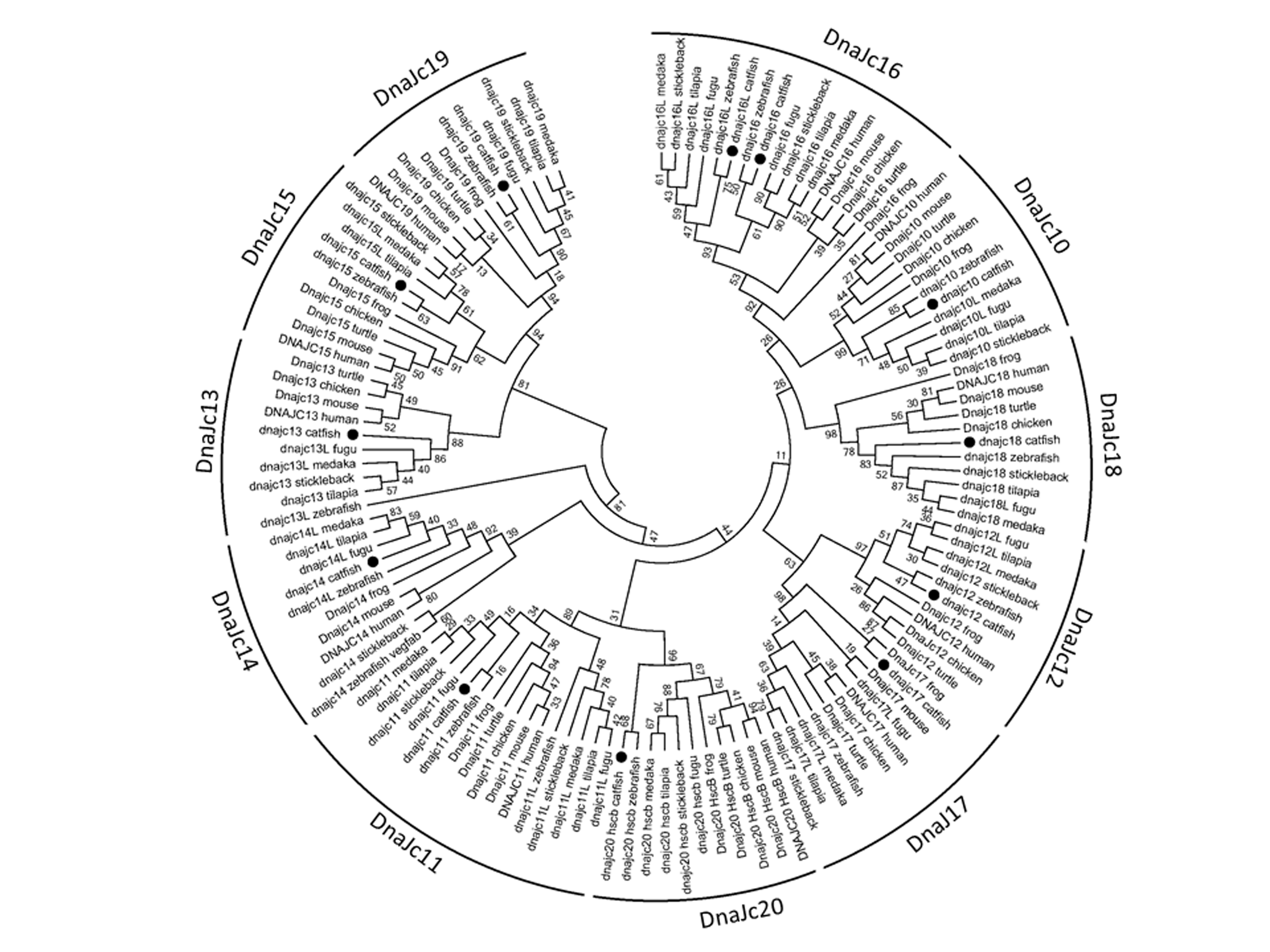

Supplement: S4 Fig — Phylogenetic tree of Hsp40s type III: Dnajc10 to Dnajc20. The phylogenetic tree was constructed as in S1 Fig. Accession numbers for all sequences are provided in S1 Table. The black dots indicate catfish Dnajc genes. Suffix “L” indicated “-like”. (TIF) [file pone.0115752.s004.tif]

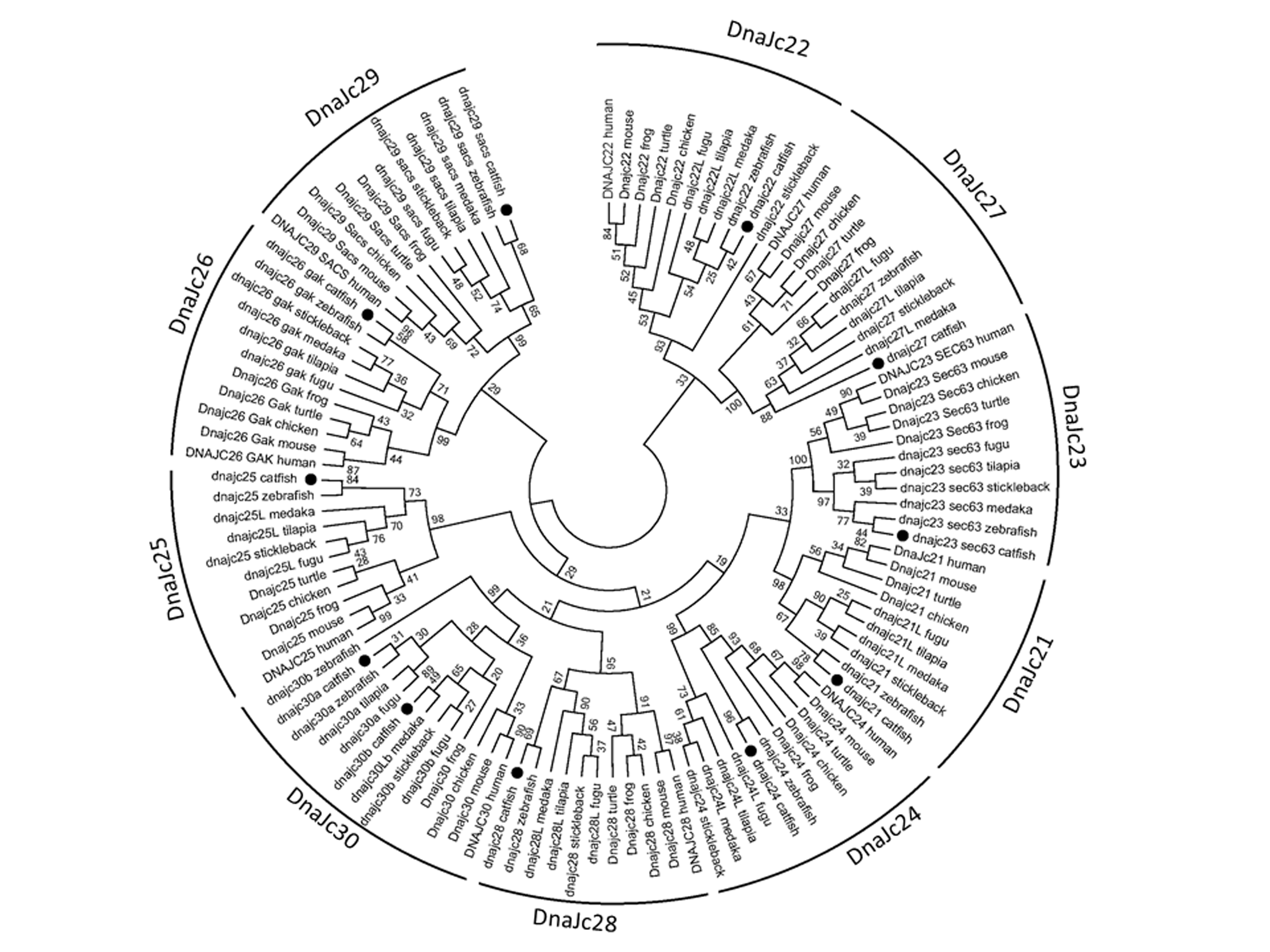

Supplement: S5 Fig — Phylogenetic tree of Hsp40s type III: Dnajc21 to Dnajc30. The phylogenetic tree was constructed as in S1 Fig. Accession numbers for all sequences are provided in S1 Table. The black dots indicate catfish Dnajc genes. Suffix “L” indicated “-like”. (TIF) [file pone.0115752.s005.tif]
